# Metabolomic profiles of induced pluripotent stem cells derived from patients with rheumatoid arthritis and osteoarthritis

**DOI:** 10.1186/s13287-019-1408-5

**Published:** 2019-11-15

**Authors:** Juryun Kim, Sunyoung Christina Kang, Na Eun Yoon, Yena Kim, Jinhyeok Choi, Narae Park, Hyerin Jung, Byung Hwa Jung, Ji Hyeon Ju

**Affiliations:** 1 CiSTEM Laboratory, Catholic iPSC Research Center, College of Medicine, The Catholic University of Korea, Seoul, 137-701 South Korea; 2 Department of Life Sciences, McMaster University, 1280 Main St W, Hamilton, Canada; 3 Molecular Recognition Research Center, Korea Institute of Science and Technology, Seoul, 02792 Republic of Korea; 4 Division of Bio-Medical Science & Technology, KIST School, Korea University of Science and Technology, Seoul, 02792 Republic of Korea; 5 Division of Rheumatology, Department of Internal Medicine, Seoul St. Mary’s Hospital, College of Medicine, The Catholic University of Korea, Seoul, 137-701 Republic of Korea

**Keywords:** Metabolomics, Rheumatoid arthritis, Osteoarthritis, Induced pluripotent stem cells, Fibroblast-like synoviocytes, Nicotinamide, Tannic acid

## Abstract

**Background:**

Metabolomics is the systemic study of the unique fingerprints of metabolites involved in cellular processes and biochemical reactions. The metabolomic approach is useful in diagnosing and predicting the development of rheumatoid arthritis (RA) and osteoarthritis (OA) and is emerging as a useful tool for identifying disease biomarkers. The aim of this study was to compare the metabolic blueprint of fibroblast-like synoviocyte (FLS) cells and induced pluripotent stem cells (iPSCs) derived from RA and OA patients.

**Methods:**

Somatic cells of RA patients (*n* = 3) and OA patients (*n* = 3) were isolated, transduced with a lentiviral plasmid, and reprogrammed into iPSCs displaying pluripotency. Metabolic profiling of RA and OA patient–derived FLS cells and iPSCs was performed using liquid chromatography/mass spectrometry and statistical analysis. After normalization by the sum of the peak intensities through LC/MS, 37 metabolites were detected across RA and OA patients.

**Results:**

The metabolites of RA and OA were distinguishable according to the PLS-DA analysis. LysoPC (20:4), 4-methoxychalcone, phosphorylcholine, and nicotinamide (NAM) were significantly higher in RA iPSCs than in OA iPSCs (*p* < 0.05). The NMNAT-3 enzyme, which catalyzes an important step in the biosynthesis of NAD^+^ from adenosine triphosphate, was also upregulated in RA iPSCs. Interestingly, the proliferation of RA iPSCs was significantly greater than OA iPSC proliferation (*p* < 0.05). NAM played a critical role in the proliferation of RA iPSCs but not in OA iPSCs. When iPSCs were treated with 100 nM of the NAM inhibitor tannic acid (TA), the proliferation of RA iPSCs was significantly reduced (*p* < 0.001).

**Conclusions:**

The metabolites of RA and OA FLS cells and RA and OA iPSCs were all clearly distinguishable from each other. NAM played a critical role in the proliferation of RA iPSCs but not in OA iPSCs. TA effectively inhibited the expression of NAM in RA iPSCs and is a possible effective treatment for RA patients.

## Background

Metabolism is the set of life-sustaining processes that are vital to cell function. Metabolomics is the quantitative measurement of metabolites, which are small-molecule intermediates and the products of metabolism [1, 2]. Metabolic analysis offers a snapshot of intermediates involved in cellular processes and physiological changes, providing an extensive understanding of the patient’s disease state [3, 4]. Metabolomics is an excitedly growing field that identifies markers for diagnosis, prognosis, and treatment of various diseases, including rheumatic disorders [5].

Rheumatoid arthritis (RA) is a chronic, progressive, autoimmune disease characterized by synovial hyperplasia and inflammation leading to swelling and pain around the joints [6, 7]. In contrast, osteoarthritis (OA) is a progressive, degenerative joint disease characterized by the narrowing of joint spaces and a wear-and-tear damage on the cartilage [8, 9]. Although the pathogenesis and mechanism of these two diseases vastly differ, the early RA disease state is difficult to discriminate from OA and other forms of arthritis. Accordingly, metabolomics can be used as reliable biomarkers for clinical diagnosis of rheumatoid disorders and improvements in clinical interventions [10, 11].

Fibroblast-like synoviocyte (FLS) cells are specialized cells in the synovium of the joints that are involved in the pathogenesis of RA [12]. It has been widely reported that RA FLS have tumor-like features and a rapid proliferation similar to cancer cells [13]. The hallmarks of RA FLS include active proliferation, migration, invasion, and proinflammatory mediator production [14]. In this study, we reprogrammed FLS cells into induced pluripotent stem cells (iPSCs) through a lentiviral vector containing Yamanaka factors. For iPSCs are capable of differentiating into cell types of all three germ layers [15], they have been applied to simulate the developmental progression of various diseases [16–18].

iPSCs can be used in the context of differential diagnosis between RA and OA iPSCs as iPSCs have the potential to model diseases and be applied to clinical settings [19]. Since iPSCs have self-renewal abilities and are pluripotent, they can be used for modeling various diseases including cardiovascular, genetic, and neurological diseases [20]. In addition, patient-derived iPSCs (such as the ones that were used in this study) can provide us with further insight into the pathogenesis and the pathophysiology of diseases [19]. Because RA has a complex disease mechanism, iPSCs can further provide insights to the disease pathophysiology that could be useful in the differential diagnosis between RA and OA, as it has been shown that patient-derived iPSCs have already been translated to clinical settings and disease discovery [21, 22]. If we can further understand the pathogenesis and the pathophysiology of RA and OA using patient-specific cells, iPSCs can be used to enhance their diagnosis. The iPSCs from RA patients could also be extended to a regeneration therapy as they have the ability to differentiate into mature chondrocytes and osteocytes, which synthesize the cartilage and bone respectively [23]. Therefore, iPSCs can be applied to clinical settings and are increasingly viewed as future prospects in regenerative therapy for future treatments of RA and OA.

There have been various studies comparing the metabolic differences between RA and OA patient–derived FLS cells, synovium fluid, and the serum [24–28] but very few studies that analyze the metabolic profile of RA and OA iPSCs. The metabolic analysis of iPSCs can be useful in discriminating the early development of RA and OA in patients [29], as their differentiation and reprogramming abilities are a more suitable model for clinical interventions of the disease than FLS [30].

In this study, we investigate the metabolic fingerprint of RA and OA patient–derived iPSCs using liquid chromatography/mass spectrometry (LC/MS) and compare them to RA and OA FLS cells. Through screening, we focused on nicotinamide (NAM) and examined its role in the proliferation of iPSCs. NAM occurs naturally as a component of biological systems and plays a crucial role in metabolic pathways and energy synthesis processes [31–33]. Furthermore, we assess the metabolic profiling in RA and OA patients and how NAM affects cell proliferation.

## Methods

### Generation of iPSCs from FLS cells and their maintenance

In a previous study, we generated a virus supernatant (SN) with reprogramming factors (OCT4, SOX2, KLF4, and c-MYC) from 293 T cells [23].

RA and OA FLS cells were cultured in Dulbecco’s medium (Gibco) with 10% fetal bovine serum (FBS) and 1% penicillin and streptomycin (P/S). The cells were maintained in a 37 °C, 5% CO_2_ incubator. 3 × 10^4^ RA and OA FLS were seeded in a six-well plate and infected with lentivirus the next day. The cell culture medium was changed daily until the iPSC colonies developed. Colonies were picked and expanded. RA iPSCs (*n* = 3) and OA iPSCs (*n* = 3) were maintained on a vitronectin-coated dish with E8 medium and Rock inhibitor in a 37 °C, 10% CO_2_ incubator.

### Alkaline-phosphatase staining

For alkaline-phosphatase staining, iPSCs were cultured at low density for 5 days before staining. The cells were washed with 1 mL of PBS and fixed in 1 mL of 4% paraformaldehyde at room temperature for 2–5 min. The cells were washed twice with PBS, and then 1 mL of the staining solution was added in a 2:1:1 ratio of Fast Red Violet, Naphthol AS-BI phosphate solution, and water (alkaline-phosphatase detection kit, Millipore). The cells were incubated in the dark at room temperature for 15 min. The cells were rinsed once with a TBST buffer solution (20 mM Tris-HCl, pH 7.4, 0.15 M NaCl, and 0.05% Tween-20) and twice with PBS.

### Immunofluorescence assay

The cells were washed twice with PBS and fixed with 1 mL of 4% paraformaldehyde for 30 min. After washing, the cells were incubated for 10 min at room temperature using 1 mL of NH_4_Cl solution. The cells were permeabilized using 0.1% Triton X-100 for 10 min and blocked for 30 min at room temperature in PBS containing 2% bovine serum albumin (Sigma-Aldrich) (PBA). Consequently, the primary antibodies OCT4 (Santa Cruz Biotechnology, 1:100 dilution), SSEA-4 (EMD Millipore, 1:200), TRA-1-60 (EMD Millipore, 1:200), SOX2 (BioLegend, 1:100), TRA-1-81 (EMD Millipore, 1:100), KLF4 (Abcam, 1:250), and NMNAT3 (Santa Cruz Biotechnology, 1:100) were diluted with PBA and the cells were incubated for 2 h at room temperature. After washing with PBA, the cells were incubated with Alexa Fluor 594-conjugated or 488-conjugated secondary antibodies (Life Technologies) in the dark for 2 h. For staining the nuclei, 4′,6-diamidino-2-phenylindole was incubated for 20 min at room temperature. The cells were mounted using ProLong Antifade mounting reagent (Thermo Fisher Scientific) and analyzed by Leica immunofluorescence microscopy.

### Metabolite extraction for LC/MS

Ice-cold 70% methanol (120 μL) was added to the cell pellets, and the solution was vortexed for 1 min. Cell pellets were lysed by three consecutive freeze/thaw cycles using liquid nitrogen, and the lysates were centrifuged for 10 min at 20,817*g* (14,000 rpm). The resulting supernatant was transferred to a clean vial, and 10 μL was injected into an Ultimate 3000 UHPLC system-LTQ Orbitrap Velos ProTM mass spectrometer (Thermo Scientific, San Jose, CA, USA).

### Measurement of LC/MS

Cellular metabolic profiling was performed using an Ultimate 3000 UHPLC system consisting of an autosampler and a column oven coupled to an LTQ Orbitrap Velos ProTM mass spectrometer (Thermo Scientific, San Jose, CA, USA). An ACQUITY UPLC HSS T3 column (2.1 × 100 mm, 1.8 μm; Waters) was maintained at 40 °C. Gradient elution was carried out at a flow rate of 0.4 mL min^−1^ using mobile phase A (0.1% formic acid in distilled water) and mobile phase B (0.1% formic acid in methanol). After maintaining initial conditions of 99% A and 1% B (v/v) for 2 min, a linear gradient that reached 100% B over 14 min was applied, followed by holding for 1 min at 100% B. The column was then re-equilibrated at initial conditions for 3 min. The autosampler was kept at 4 °C throughout the analysis. All samples were analyzed randomly to eliminate the effects of analysis order. MS using an electrospray ionization source was operated in both positive and negative ionization modes. The capillary voltages of positive and negative modes were + 3.2 kV and 2.5 kV, and the cone voltage was 40 V for both polarities. MS spectra were analyzed at a mass range of 50–1200 Da in the data-independent centroid mode (MS^E^ resolution). A quality control (QC) sample, prepared by pooling equal volumes of each sample, was used for column conditioning, which was performed by injecting 10 times before analytical runs. The QC sample was also analyzed every 10 analytical sample runs to evaluate the repeatability of the instrument.

### Data processing

The obtained metabolic raw data were normalized by the sum of peak intensity of all detected ions and each cell number. Partial least squares regression (PLS-DA) statistics were performed with SIMCA-P software (Umetrics AB, UMEÅ, Sweden). To acquire metabolite identification, the online databases (METLIN: http://metlin.scripps.edu, HMDB: http://www.hmdb.ca, and MassBank: http://www.massbank.jp) were used to identify metabolites using exact mass and MS/MS value. After searching these databases and comparing the mass fragment patterns of the references, the identified metabolites were cross-checked with commercially available validated standards. Due to the structural similarity of fragmentation patterns of lipids such as phosphatidylcholines and lysophosphatidylcholines (LysoPC), we implemented the strategies reported by [34, 35].

Statistical significance of each group in metabolic changes was evaluated by a univariable *t* test. *p* values less than 0.05 were considered to be statistically significant, and *p* values were based on logarithmic transformed data with Pareto scaling.

### Inhibition of the enzyme NMNAT 3 using tannic acid and STF-118804

To confirm the function of the NMANT 3 enzyme, RA and OA patient–derived iPSCs were treated with 100 nM and 200 nM of tannic acid (TA) and 1 nM and 2.5 nM of STF-118804. TA and STF-118804–treated iPSCs were incubated for 24 h.

### Cell-counting kit assay

RA and OA iPSCs were seeded onto 96-well plates and incubated for 24 h. The following day, each well was treated with tannic acid and STF-118804 and then incubated for 1–4 h with 10 μL/well of Cell Counting Kit-8 (Dojindo). Absorbance at 450 nm was measured using a microplate reader (VersaMax).

### RNA extraction for RT PCR

RNA of iPSCs and FLS cells was extracted using Trizol (Invitrogen). We synthesized cDNA from RNA through RevertAid™ First Strand cDNA Synthesis kit (Thermo Fisher Scientific). We performed reverse transcriptase (RT) polymerase chain reactions (PCRs) using the synthesized cDNA synthesized from iTaq DNA Polymerase Kit (iNtRON Biotechnology). Quantitative real-time PCR was performed with the LightCycler® 480 instrument (Roche), the SYBR Green Real-time PCR Master Mix (Roche). Gene expression levels were normalized to GAPDH expression levels. The primer sequences are presented in Additional file 5: Table S1.

### siRNA transfection

After seeding 4.0 × 10^3^, 1.0 × 10^5^ cells of OA and RA iPSCs on 6-well and 96-well plates, transfection of siRNA control and siRNA against NMNAT3 was performed. 7.5 μL of Lipofectamine 3000 reagent (Thermo Fisher Scientific) was added to 125 μL of Opti-MEM Reduced Serum Medium. In another tube, siRNA 5 nM was added and treated to another 125 μL of Opti-MEM Reduced Serum Medium. The two tubes were mixed and then incubated at room temperature for 10 min. After changing to a fresh E8 media on a plate with the cells, the siRNA solution was added. Two days later, RNA was extracted, followed by real-time PCR after cDNA synthesis as described above. Relative mRNA levels of NMNAT3 and Proliferating Cell Nuclear Antigen (PCNA) gene were compared, and the primer sequences were added to the table (Additional file 1: Table S1). The siRNA sequence used here is as follows.

Negative control: sequence (5′-3′) UUC UCC GAA CGU GUC ACG UTT, ACG UGA CAC GUU CGG AGA ATT

siRNA against NMNAT3: sequence (5′-3′) GGAUGCACCAGCACAACAUTT,  AUGUUGUGCUGGUGCAUCCTT

In the case of the 96 well, siRNA was transfected as described above, and 2 days after seeding, proliferation assay was performed using the CCK-8 kit.

### Annexin V assay

After seeding OA and RA iPSCs, they were treated with 0 and 100 nM of tannic acid and harvested after 2 days. After washing once with PBS, it was washed with a binding buffer of Annexin V Apoptosis Detection Kit APC (Thermo Fisher Scientific). Five microliters of APC-conjugated Annexin V was added and incubated for 15 min at room temperature. After washing with the binding buffer, 5 uL of propidium iodide staining solution was added and cells were incubated at room temperature for 10 min and washed with Binding Buffer. Cell populations were analyzed using BD LSR Fortessa flow cytometry (BD Bioscience).

### Mitochondria stress test

OA and RA iPSCs were seeded on a vitronectin-coated XF-analyzer 24-well microplate at 5 × 10^4^ cells and treated with 10 μM Rock inhibitor. The E8 media was changed daily for 2 days and incubated at 37 °C for 2 days. Using an Agilent Seahorse XF cell Mito stress test kit (Agilent Technology, CA, USA), Oligomycin 1 μM, FCCP 0.5 μM, and Rotenone + Antimycin A 0.5 μM were treated into OA and RA iPSCs by the manufacturer’s protocol and the oxygen consumption rate (OCR) was measured using Seahorse XF analyzer (Agilent Technology, CA, USA). A mean value was calculated for each OA and RA iPSC group.

### Statistical analysis

Statistical analyses were performed using GraphPad Prism software. The results are shown as the mean and the standard error of the mean. Error bars represent the standard error of the mean. Differences between groups were analyzed for statistical significance using Student’s *t* test or an analysis of variance (ANOVA) test. The *t* test was applied to analyze nonparametric quantitative datasets, and a two-tailed *p* value was calculated, with *p* < 0.05 indicating statistical significance.

## Results

### Generation of disease-specific iPSCs from FLS

**Fig. 1:**
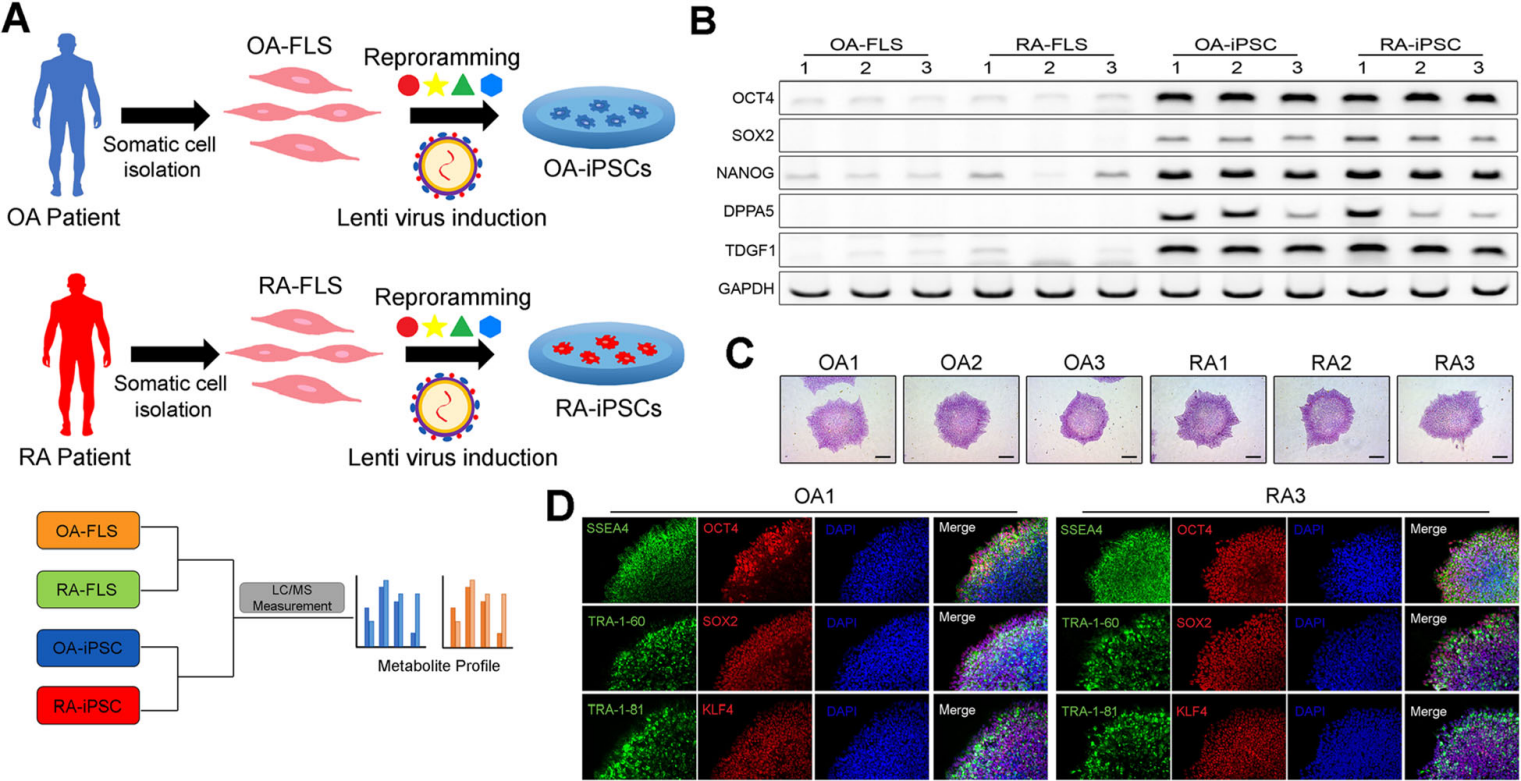
iPSC generation from OA and RA patient FLS cells. **a** Scheme of iPSC generation from OA and RA patient FLS cells and LC-mass measurement. **b** RT PCR data of iPSC pluripotent markers. **c** Alkaline phosphatase staining of OA and RA patient iPSCs. **d** Immunofluorescence assay data of OA and RA patient iPSCs

Somatic cells of RA patients (*n* = 3) and OA patients (*n* = 3) were isolated, transduced with a lentiviral plasmid, and reprogrammed into iPSCs displaying pluripotency (Fig. 1a and Additional file 1: Figure S1). FLS cells and iPSCs of RA and OA patients were analyzed using LC/MS. To ensure the established iPSCs were pluripotent, we performed RT PCR to investigate the expression of pluripotent markers. After comparing gene marker expression for OCT4, SOX2, NANOG, DPPA-5, and TDGF1, we confirmed that the genes that were faintly or not expressed in FLS cells were well expressed in RA and OA iPSCs after reprogramming (Fig. 1b). RA and OA iPSCs were successfully stained with alkaline phosphatase, demonstrating their undifferentiated state (Fig. 1c). Immunofluorescence analysis confirmed that the iPSC markers, namely SSEA4, OCT4, TRA-1-60, SOX2, KLF4, and TRA-1-81, were expressed (Fig. 1d). Thus, we confirmed that the iPSCs derived from RA and OA FLS were pluripotent.

### Principal component analysis of RA and OA patient-derived FLS and iPSCs

**Fig. 2:**
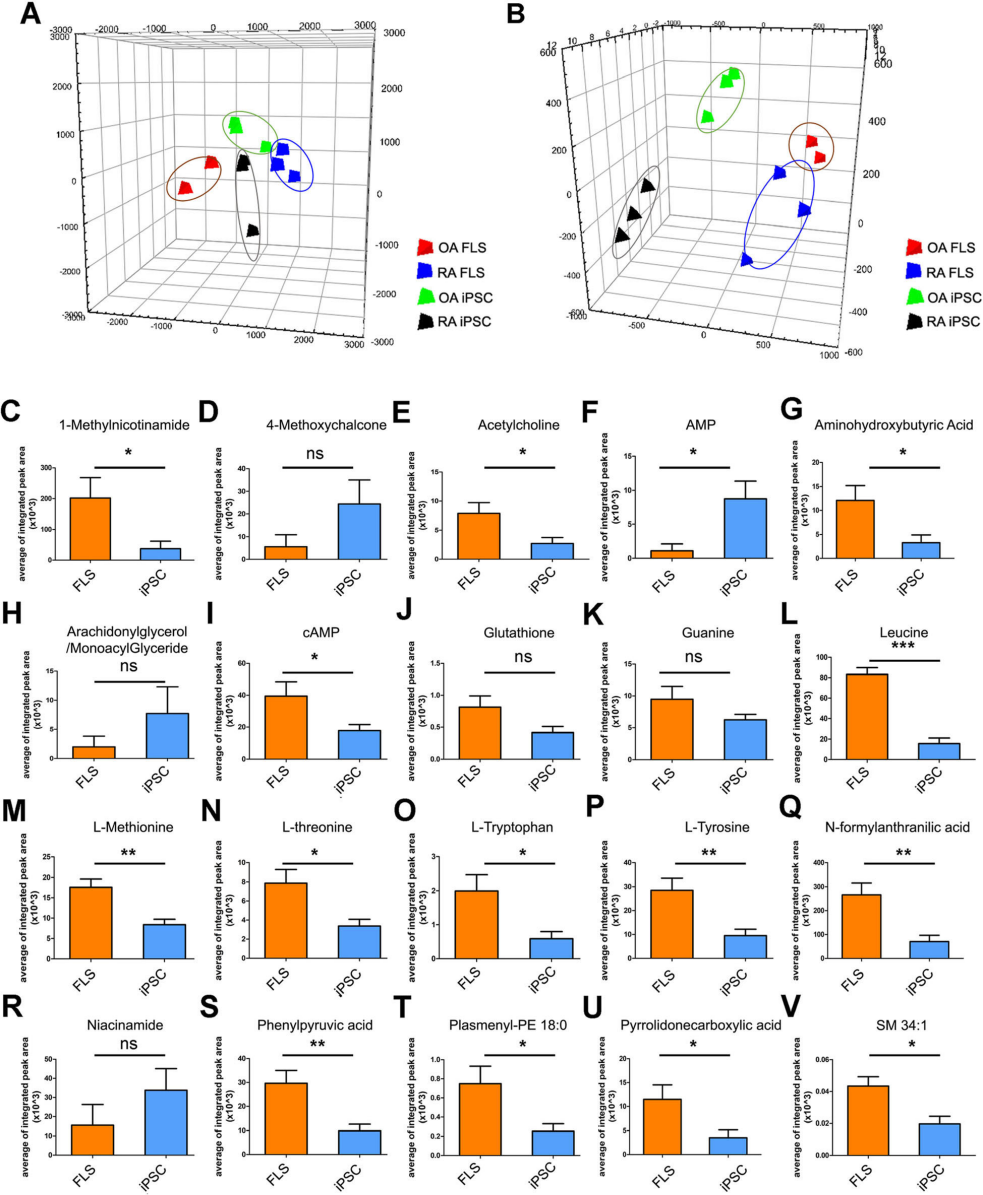
Multivariate statistical analysis based on metabolomic profiling data to compare FLS cells with iPSCs derived from OA and RA patients. PLS-DA scores plot of **a** positive mode and **b** negative mode. **c**–**v** Significantly altered metabolites were shown in both groups when reprogramming FLS to iPSC. Data presented mean ± SEM. All data was analyzed by Student’s *t* test. *p < 0.05, **p < 0.01, ***p < 0.001

After extracting the metabolites through LC/MS, we conducted a PLS-DA analysis between FLS cells and iPSCs of RA and OA patients (Fig. 2). The metabolites of RA and OA were distinguishable according to the PLS-DA analysis. When comparing the metabolites of RA FLS cells with OA FLS cells, the PLS-DA analysis shows a clear distinction between the two clusters in positive mode and negative mode (Fig. 2a, b). The metabolites of RA iPSCs and OA iPSCs appeared in different clusters and were clearly distinguishable, indicating that there was a difference in their metabolic profiles. RA and OA iPSC metabolites were also distinguishable from RA and OA FLS cells. Therefore, we confirmed that the metabolites of the parental cells FLS go through metabolic alterations when reprogrammed into iPSCs.

### Identification of metabolites using LC/MS

After normalization by the sum of the peak intensities through LC/MS, we detected a total of 37 metabolites across RA and OA patients. All metabolites and their associated metabolic pathways are summarized in Additional file 5: Tables S2, S3, S4, and S5. Out of 37 metabolites which were tested, 13 metabolites were found to be higher in RA FLS than OA FLS, including nicotinamide, lysophosphatidylcholine (lysoPC; 20:4), adenine, and adenosine monophosphate (Additional file 5: Table S2). Thirteen metabolites were more upregulated in OA FLS than in RA FLS cells, which involved proline, glutamic acid, and aspartic acid. However, the average of the integrated peak area showed only modest differences (Additional file 5: Table S2).

**Fig. 3:**
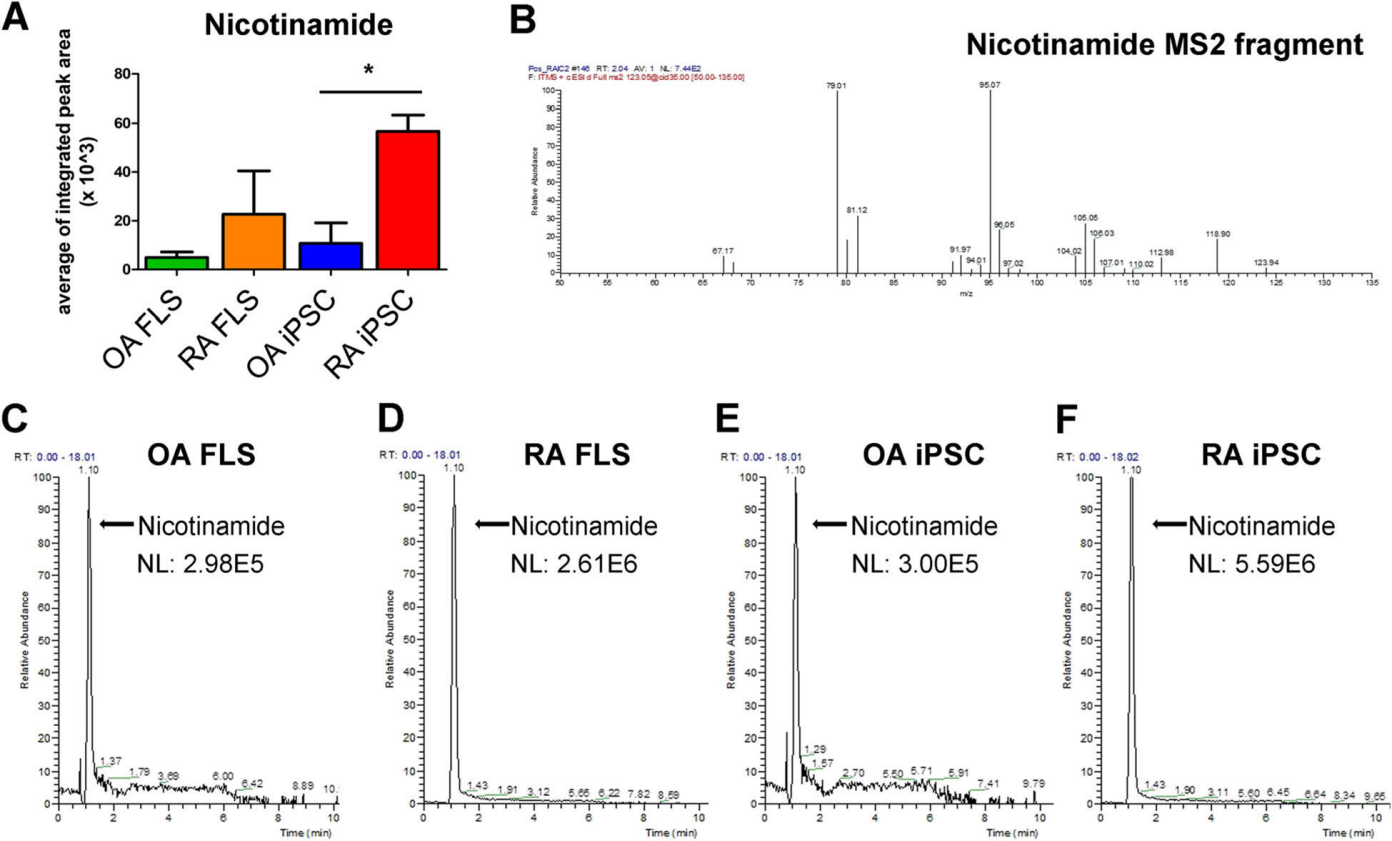
Analytic LC-mass data of OA FLS, RA FLS, OA iPSC, and RA iPSCs. **a** Integrated peak area of nicotinamides was shown in the OA, RA FLS group and the OA, RA iPSC group. Data presented mean ± SEM. All data was analyzed by Student’s *t* test. *p < 0.05, **p < 0.01, ***p < 0.001. **b** Fragment mass peak of nicotinamide. **c**–**f** Mass peak histogram of nicotinamide in the OA, RA FLS group and the OA, RA iPSC group. Each NL expressed peak area

Figure 2c summarizes the metabolites that were detected at higher levels in FLS than in iPSCs of both the RA and OA groups combined. 1-Methylnicotinamide, acetylcholine, aminohydroxybutyric acid, cAMP, leucine, l-methionine, l-threonine, l-tryptophan, l-tyrosine, N-formylanthranilic acid, phenylpyruvic acid, plasmenyl-PE 18:0, pyrrolidonecarboxylic acid, and SM 34:1 were significantly higher in FLS cells than in iPSCs (*p* < 0.05, Fig. 2c). In particular, adenosine monophosphate (AMP) was significantly elevated in iPSCs, by 7.92-fold compared to FLS cells. Arachidonylglycerol, 4-methoxychalcone, and NAM were detected at up to 3.85-, 4.36-, and 2.16-fold greater levels in iPSCs than in FLS cells, respectively (Additional file 5: Table S3). Eighteen out of 26 metabolites, including nicotinamide, 4-methoxychalcone, and lysoPC, were higher in RA iPSCs than in OA iPSCs (Additional file 5: Table S4). When comparing RA iPSC and OA iPSC metabolites, nicotinamide, 4-methoxychalcone, and lysoPCs were significantly higher in RA iPSCs than in OA iPSCs (Additional file 2: Figure S2 and Fig. 3a).

### Nicotinamide was expressed more in RA iPSCs than in OA iPSCs

Metabolite differences of nicotinamide between FLS cells and iPSCs were found to be significantly different (*p* < 0.05) from OA and RA iPSCs (Fig. 3a). This was confirmed by LC/MS spectrometry where the mass fragment peak of nicotinamide was analyzed to identify nicotinamide (Fig. 3b). Mass chromatograms of nicotinamide revealed that there was a greater difference in spectrum peak area between OA iPSCs (NL = 3.00E5) and RA iPSCs (NL = 5.59E6) than between OA FLS (NL = 2.98E5) and RA FLS (NL = 2.61E6) (Fig. 3c–f).

### NMNAT 3 gene expression in RA iPSCs

**Fig. 4:**
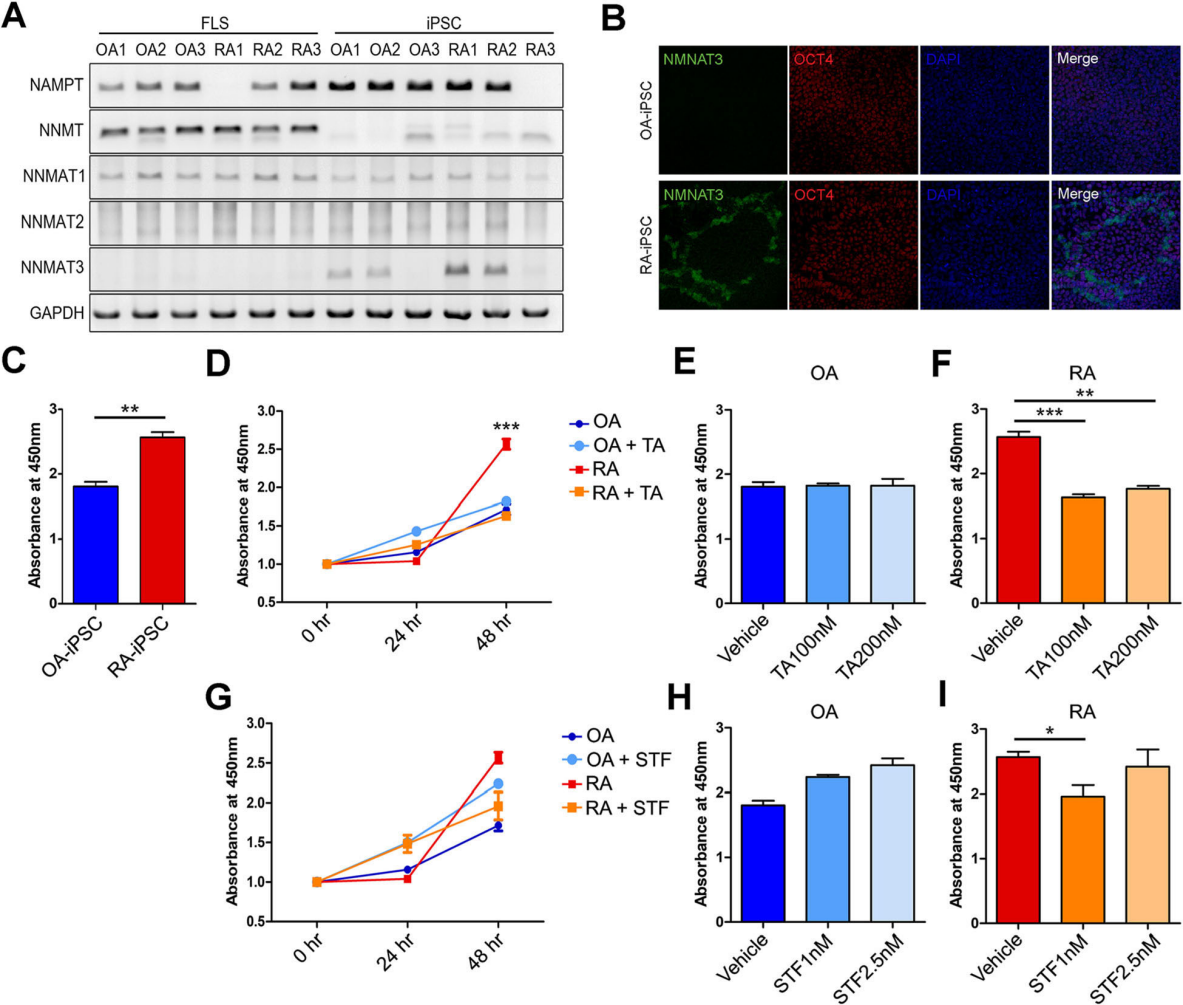
Proliferation assay of OA and RA iPSC (**a**) RT PCR data expressed as mRNA levels of markers related to salvage pathways. **b** Immunofluorescence assay of NMNAT3. **c** CCK-8 assay of OA and RA iPSCs expressed as the difference of proliferation. **d** CCK-8 assay of OA and RA iPSCs showing the difference of proliferation after treatment with tannic acid (TA), inhibitor of NMNAT3. **e** CCK-8 assay data of OA iPSCs after treatment with TA. **f** CCK-8 assay data of RA iPSCs after treatment with TA. **g** CCK-8 assay of OA and RA iPSCs showing some difference of proliferation after treatment with STF-118804, inhibitor of NAMPT. **h** CCK-8 assay data of OA iPSCs after treatment with STF. **i** CCK-8 assay data of RA iPSCs after treatment with STF. Data presented mean ± SEM. All data was analyzed by the Student *t* test. *p < 0.05, **p < 0.01, ***p < 0.001

To understand the contribution of intracellular NAD^+^ on cell proliferation, we monitored the changes in gene expression for the enzymes catalyzing NAD+ biosynthesis. We investigated mRNA levels of NAMPT, NMNAT 1, NMNAT 2, and NMNAT 3, which are rate-limiting enzymes in the NAD^+^ salvage pathway. The expression of NMNAT 3 was significantly upregulated in RA iPSCs compared with OA iPSCs (Fig. 4a). Although the expression of NAMPT was more pronounced in iPSCs than in FLS, the difference was not statistically significant between RA and OA. An immunofluorescence assay confirmed that the expression of the NMNAT 3 gene increased more in RA iPSCs than in OA iPSCs (Fig. 4b).

### Proliferation assay of RA iPSCs and OA iPSCs

Cell proliferation was significantly more elevated in RA iPSCs than OA iPSCs (*p* < 0.01, Fig. 4c, Additional file 3: Figure S3a). To confirm the function of NMNAT 3 in cell division and proliferation, a proliferation assay was performed on the inhibitor-treated RA and OA iPSCs. We determined the effects of depletion of intracellular NAD^+^ pools by treatment with TA and STF-118804, which are specific inhibitors of NMNAT 3 and NAMPT, respectively [36–38].

In this study, we have treated RA and OA iPSCs using TA concentrations of 100 nM and 200 nM. We have attempted to use higher concentrations of tannic acid, 500 nM and 10 μM, to examine its effect on proliferation, but these concentrations were too toxic for the cells as the cells did not survive 24 h after treatment. Upon treatment, the morphology of iPSCs was abnormally changed at 500 nM and cell death was observed at 10 μM. Thus, proliferation results were obtained in TA at 0-, 100-nM, and 200-nM ranges, since the cell survival as well as the proliferation were sustained upon 48 h after treatment. Therefore, we believe that such concentrations do not simply induce cell death, but actually poses the ability to reduce the proliferation of cells.

By treatment with TA at 48 h, RA iPSC proliferation diminished significantly compared with the vehicle control (*p* < 0.001, Fig. 4d). The proliferation of RA iPSCs dropped significantly when they were treated with 100 nM and 200 nM TA (in the case of 100 nM, *p* < 0.001; in the case of 200 nM, *p* < 0.05, Fig. 4f). When OA iPSCs were treated with 100 nM and 200 nM TA, the proliferation did not change (Fig. 4e).

After treating three OA and RA iPSCs with tannic acid for 2 days, real-time PCR was performed. mRNA expression levels of cell proliferation markers PCNA and Ki67; cell cycle arrest markers P21, CDK4, and CDK6; apoptosis marker BAX; and anti-apoptosis marker Bcl-2 were investigated. In the RA group, PCNA and Ki67 decreased at TA 100 nM, indicating a reduction in cell proliferation (Additional file 4: Figure S4c, d). It is known that p21 becomes upregulated and CDK4 and CDK6 are cell cycle checkpoints that form complexes when cell arrest occurs [39, 40]. Because these markers were not upregulated in the RA group, it does not seem to be related to cell arrest (Additional file 4: Figure S4e-g). Moreover, apoptosis marker BAX was not increased at TA 100 nM in the RA group, but increased at TA 200 nM in the RA group (Additional file 4: Figure S4 h). Bcl-2, anti-apoptosis marker, was decreased at TA 200 nM in the RA group (Additional file 4: Figure S4i). Two days after TA 0 nM and 100 nM treatment, Annexin V staining was performed to measure the population of apoptotic cells by flow cytometry (Additional file 4: Figure S4J-Q). In the OA group, early apoptotic cells increased from 1.8% in control to 3.08% at TA 100 nM and in the RA group and apoptotic cells slightly increased from 4.25% in control to 5.17% at TA 100 nM (Additional file 4: Figure S4j). Late apoptotic cells increased slightly from 1.7% in control to 2.9% at TA 100 nM in the OA group and changed slightly from 4.3% in the control to 4.0% at TA 100 nM in the RA group (Additional file 4: Figure S4k). There was a little change to the number of viable cells (Additional file 4: Figure S4 l). In conclusion, cell proliferation may be reduced at TA 100 nM independent of cell arrest and apoptosis.

RA iPSCs treated with STF-118804 also exhibited a decrease in cell proliferation, but the reduction was not as dramatic as with TA treatment (Fig. 4g). Interestingly, the proliferation of RA iPSCs was significantly reduced with 1 nM STF-118804 but increased with 2.5 nM STF-118804 (fig. 4i). The proliferation of OA iPSCs increased when treated with both 1 nM and 2.5 nM STF-118804 but was not significant (Fig. 4h).

The siRNA against NMNAT 3 was transfected, and 2 days later, mRNA expression of NMNAT 3 was determined by real-time PCR. Relative gene expression was significantly reduced in RA iPSCs and OA iPSCs with siRNA against NMNAT 3 compared to the control (Additional file 3: Figure S3b). As a result, it was confirmed that the gene knocked down using siRNA against NMNAT3. In addition, the relative gene expression of Ki67, a proliferation marker, was measured by real-time PCR. Ki67 levels were decreased in the RA group transfected with siRNA against NMNAT3 compared to the RA group transfected with negative control against siRNA (Additional file 3: Figure S3c).

Next, 4.0 × 10^3^ cells of OA and RA iPSCs were seeded on a 96-well plate, and siRNA against NMNAT3 were transfected. Two days after seeding, a proliferation assay was performed. As a result, it was observed that the O. D value at absorbance 450 nm was reduced to 6.25% in the OA iPSC group compared with the negative control against siRNA and 11.8% in the RA iPSC group treated with the siRNA against NMNAT3 (Additional file 3: Figure S3d). This result shows that the proliferation also decreases when the NMNAT3 gene is reduced.

These results suggest that the cell cultures with NAM significantly improve the proliferation of reprogrammed iPSCs. Our data strongly demonstrates that NAM facilitates the proliferation of RA iPSCs by enhancing the generation of adenosine triphosphate (ATP).

### Different mitochondrial function in OA and RA iPSCs

**Fig. 5:**
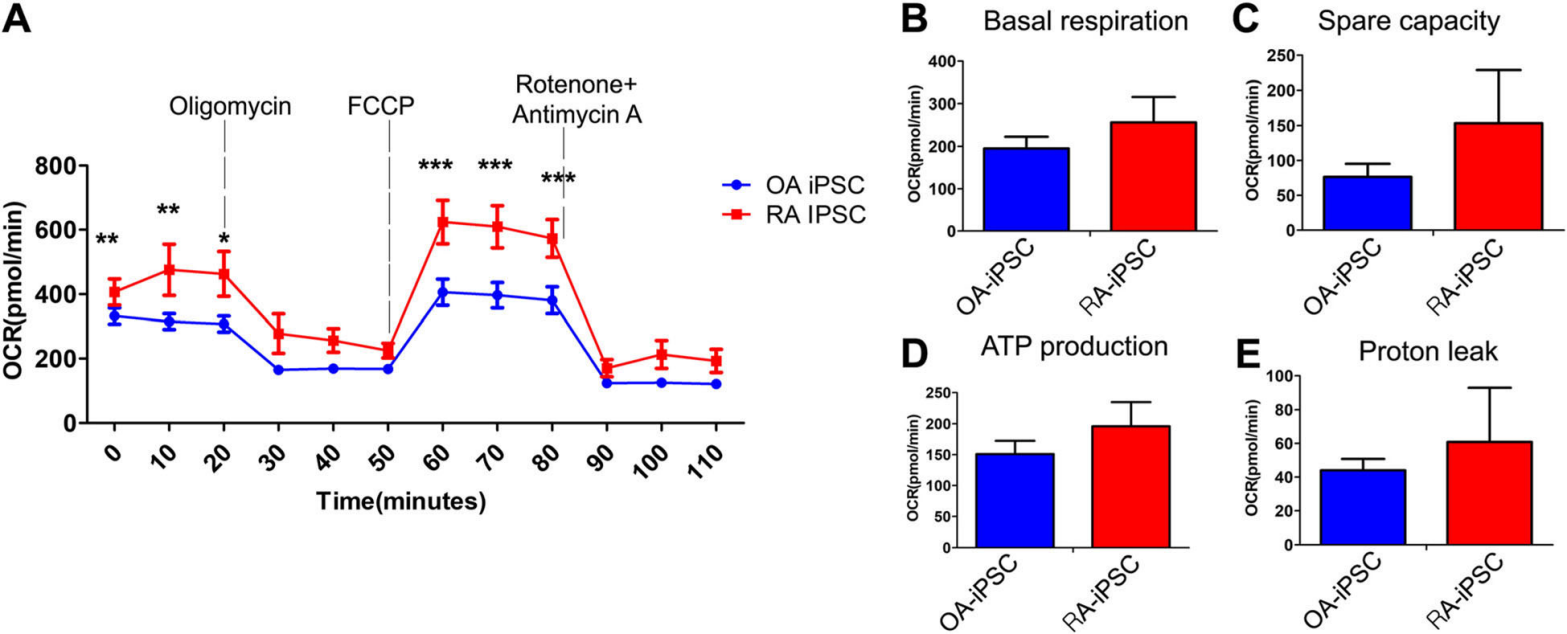
A mitochondrial function assay of OA and RA iPSCs. **a** Oxygen consumption rate (OCR) of OA and RA iPSCs was measured by a Seahorse XF analyzer. Oligomycin, FCCP, and Rotenone + Antimycin A were treated at the indicated point. **b** Statistical analysis of basal respiratory in the OCR curve. **c** Statistical analysis of spare respiratory capacity level in the OCR curve. **d** Statistical analysis of ATP production levels in the OCR curve. **e** Statistical analysis of proton leak level in OCR curve. OCR curve was presented as mean of OA iPSCs (*n* = 3) and RA iPSCs (*n* = 3). Data presented mean ± SEM. All data was analyzed by ANOVA test. *p < 0.05, **p < 0.01, ***p < 0.001

A mitochondrial stress assay of OA and RA iPSCs was performed using an XF-analyzer to confirm the differences in mitochondria function. As a result, the OCR of RA iPSCs was higher than that of OA iPSCs (Fig. 5a). After FCCP processing, it was found that RA iPSCs presented with a significantly higher OCR change than OA iPSCs. In addition, the mean value of basal level (OA group 195 pmol/min, RA group 256.8 pmol/min), spare repository capacity (OA group 76.5 pmol/min, RA group 153.5 pmol/min), ATP production (OA group 151 pmol/min, RA group 195.9 pmol/min), and proton leak level (OA group 44.1 pmol/min, RA group 60.9 pmol/min) were all higher in RA iPSCs (Fig. 5b–e). This result indicated that the metabolic difference is due to differences in mitochondrial function.

## Discussion

The aim of this study was to compare RA and OA iPSC metabolites and contrast them to RA and OA FLS cells. To date, the vast majority of published studies investigated the metabolites of RA and OA FLS of the serum and synovial fluid [41–43]. Although the pathogenic behaviors and metabolomics of RA and OA FLS have been studied extensively, the metabolic profiles of RA and OA iPSCs have not yet been explored. The differences in metabolites between RA and OA iPSCs have not been reported to our knowledge. Our study shows that the metabolic profile of RA-patient iPSCs is discrete not only from the profile of the OA-patient iPSCs, but also from FLS. Specifically, principal component analysis showed that there is a difference in the metabolites between RA and OA FLS (Fig. 2a), suggesting that the metabolic phenotype of RA FLS cells is considerably different from that of quiescent OA FLS cells [44]. This finding is supported by the fact that the RA FLS is phenotypically different from OA FLS in that RA FLS have a high proliferation rate and tumor cell-like characteristics that contribute to synovial hyperplasia and inflammation.

According to the LC/MS, glutamic acid and proline were found in greater amounts in OA FLS than RA FLS (Additional file 5: Table S2). Glutamic acid is known to play a critical role in protein synthesis and produce enzymes for the citric acid cycle and gluconeogenesis [13], and proline maintains the structure of human collagen and connective tissue [45]. The metabolites that were higher in RA FLS than OA FLS were also examined. Adenine, which is an essential metabolite involved in the production of FAD, ATP, and NAD^+^ during cellular respiration, was higher in RA FL than in OA FLS [46]. We believe that the high levels of adenine may be associated to the strong proliferation of RA FLS, as ATP controls the cell cycle and induces cell proliferation. Therefore, we believe that RA FLS will require higher levels of ATP than OA FLS for the increase in cell proliferation.

When comparing RA and OA FLS to RA and OA iPSCs, various metabolites involved in cellular processes such as energy production and immune responses were noted, namely adenosine monophosphate (AMP), 4-methoxychalcone, and arachidonylglycerol. AMP is an organic component of the energy-carrying molecule ATP that can be used for high-energy expenditure, and intracellular signaling. AMP) was significantly upregulated in iPSCs by 7.92-fold compared to FLS. Another metabolite that was noticeably higher in iPSCs than FLS was 4-methoxychalcone, which inhibits the cytokines involved in systemic inflammation such as TNFα and demonstrates anti-inflammatory activities [47, 48]. 4-Methoxychalcone was higher in iPSCs than in FLS by 4.36-fold. Arachidonylglycerol, which mediates immunomodulatory effects and reduces proinflammatory markers such as C-reactive protein (CRP), interleukin 6 and 1β, and TNFα in RA [49–51], was 3.85-fold higher in iPSCs than in FLS. Previous studies showed that it also regulates gene expression for enzymes responsible for lipid storage and fatty acid metabolism [51].

When comparing the metabolic differences between RA and OA iPSCs, lysoPC (20:4) and nicotinamide (NAM) were detected at higher levels in RA iPSCs than in OA iPSCs (Additional file 5: Table S4). LysoPC is a major class of glycerophospholipid with specific receptors involved in cellular growth and differentiation [52, 53]. It is known to be implicated in inflammation, insulin resistance, obesity, and type 2 diabetes [53]. However, the effects of Lyso PC on RA and OA are beyond the scope of this study and further research will be needed to examine its role of lysoPC on RA and OA patients.

It has been widely reported that NAM plays a critical role in cell proliferation, energy metabolism, mitochondria functions, and differentiation [54]. Furthermore, NAM increases the proliferation rate and lowers the apoptosis rate during iPSC reprogramming [15, 54]. NAM was significantly higher in RA iPSCs than in OA iPSCs (Fig. 3a), suggesting that RA iPSCs have a higher proliferation than OA iPSCs. This assumption was based on the fact that RA FLS, their somatic cells of origin, have a profoundly higher proliferation than OA FLS. By conducting a proliferation assay of RA and OA iPSCs, we have indeed confirmed that the proliferation of RA iPSCs was significantly higher than that of OA iPSCs (Fig. 4c). This highly suggests that the RA iPSCs may also have a rapid proliferation similar to RA FLS that accounts for their tumor-like characteristics.

Multiple studies claim that the phenotype of RA FLS is distinctly more aggressive than OA FLS [50, 55–57]. This may be due to the fact that there are increased levels of inflammatory cytokines, reactive oxygen species, and nuclear factors that trigger inflammation in the synovium in RA [58]. Such inflammatory response escalates resting energy expenditure and thermogenesis [55]. We found that RA iPSCs, similar to RA FLS cells, were more vigorous than OA iPSCs in their proliferation.

Studies show that cancer cells and RA FLS require plentiful nutrients to facilitate their rapid growth and proliferation [59]. One of the essential molecules that enable their active proliferation is glucose, in which glucose is converted to pyruvate to generate ATP through glycolysis [50, 57]. We believe that the high activation of glycolysis and gluconeogenesis is the driving mechanism that mediates the rapid proliferation in RA FLS as well as RA iPSCs. According to Koppenol et al., 10% higher ATP synthesis was necessary for RA FLS and cancer cells compared with the energy requirement of normal or OA FLS cells [56]. This finding is highly relevant to our data as NAM is a major intermediate of ATP synthetic pathways, including glycolysis and the citric acid cycle [41]. It is the dominant NAD^+^ precursor and involved in de novo synthesis and the salvage and Preiss Handler pathways [36]. This is supported by the fact that NMNAT 3, a major intermediate of NAD^+^ biosynthesis, was strongly expressed in RA iPSCs (Fig. 4a). NMNAT 3 catalyzes intermediates that lead to oxidation into acetyl-CoA via the tricarboxylic acid cycle to release energy [32].

In Fig. 5, various inhibitors that block the cellular respiration were induced on OA and RA iPSCs in order to see the difference in their mitochondria functions. Oligomycin inhibits oxidative phosphorylation and ATP production by blocking ATP synthase as well as reducing the electrons from flowing in the electron transport chain [60]. The RA iPSCs show a significantly higher mitochondria oxygen consumption rate than the OA iPSC upon 20 min of oligomycin treatment (Fig. 5a). This indicates that RA iPSCs have a more upregulated cellular respiration than OA iPSCs.

Rotenone and antimycin are the main potent inhibitors of the mitochondrial respiratory chain, inhibiting the complex I and the cytochrome bc1complex, respectively in the electron transport chain [60, 61]. Both of these molecules prevent the availability of oxygen for cellular respiration. Despite this, RA iPSCs showed a significantly higher OCR than OA iPSCs. We believe that this may be due to the RA iPSCs having significantly higher levels of nicotinamide (NAM) than OA iPSCs (Fig. 3a), which may have been able to surpass the level of inhibition of NADH by rotenone and antimycin.

The proliferation of RA iPSCs declined dramatically when RA iPSCs were treated with 100 nM TA (Fig. 4f, *p* < 0.001). Previous studies show that TA has an anti-tumor and anti-cancer effect by condensing the chromatin, lowering the DNA content, and inducing programmed cell death in cancer cells [62, 63].. In Serrano et al., biological properties such as antioxidant, antimicrobial, and antiviral effects are also described [64]. Along with antioxidants and anti-inflammatory and analgesic agents, we suggest that TA may be incorporated into future treatments of RA. Natural foods that contain tannic acid are green tea, red wines, and in plants and fruits such as strawberries, blueberries, apricots, mint, rosemary, and basil [65].

STF-118804 was not as effective as TA in inhibiting the proliferation of RA and OA iPSCs. This may be because TA directly inhibits the target enzyme NMNAT 3 in the final step of the NAD^+^ synthesis in the salvage pathway, whereas STF-118804 inhibits NAMPT, an enzyme in the earlier step of the salvage pathway [32, 54, 66]. We concluded that STF-118804 is not a highly effective inhibitor of NMNAT 3 and that STF-118804 may not be as effective as TA in treating RA.

Here, we suggest that nicotinamide plays a critical role in iPSCs on ATP synthesis and that TA reduces proliferation in RA iPSCs. RA iPSCs may exhibit greater ATP production and energy consumption as evidenced by extensive levels of nicotinamide (Fig. 3a) and more pronounced proliferation than was seen in OA iPSCs (Fig. 4c). TA may help reduce the excessive energy state of RA from the metabolic perspective.

This study was limited in sample size and patient profiles. The sample size was quite small and future studies need to be conducted with larger samples. Because the metabolite levels of an individual are influenced by various factors, including diet, genetics, environment, medication, and disease status, it would be worth obtaining a larger patient population with similar status. Due to the difference in the metabolic profile from individual to individual, it would be fascinating to see how the metabolites present in patients correlate with their disease and disease mechanism.

## Conclusion

The aim of this study was to evaluate the differences in the metabolic profiles of RA and OA FLS cells, as well as with RA and OA iPSCs. Our data showed that nicotinamide was highly elevated in RA iPSCs and played a crucial role in their proliferation. TA is a potential anti-inflammatory, antioxidant medication for RA patients. Because the metabolic profile for individual patients is highly dynamic, further investigation is required to validate the prevalence of these metabolites in larger RA and OA cohorts.

## Supplementary information

**Additional file 1. Fig S1.** Immunofluorescence assay of OA and RA iPSCs. Each iPSC expressed SSEA4, OCT4, TRA1-60, SOX2, TRA1-81, and KLF4, which are pluripotent markers.

**Additional file 2. Fig S2.** Statistical analysis of metabolites between OA, RA FLS cells and OA, RA iPSCs. Total of 26 metabolites including nicotinamide detected between OA, RA FLS cells and OA, RA iPSCs. Data presented mean ± SEM. All data analyzed by Student’s t-test. * means *p* < 0.05, ** means *p* < 0.01, *** means *p* < 0.001.

**Additional file 3. Fig S3.** Inhibition of NMNAT3 shows that RA iPSC proliferation was reduced. (A) Cell proliferation images on a time dependent manner. (B)(C) Real time PCR data expressed as mRNA levels of NMNAT3, Ki67 after transfection of siRNA against NMNAT3. (D) CCK-8 assay of OA and RA iPSCs showing the difference of proliferation after transfection of siRNA against NMNAT3. Real time PCR and CCK8 assay data was presented as mean of OA iPSCs (*n* = 3) and RA iPSCs (n = 3). Data presented mean ± SEM. All data was analyzed by Student t-test. * means p < 0.05, ** means p < 0.01, *** means p < 0.001.

**Additional file 4. Fig S4.** Inhibition of NMNAT3 after TA treatment. (A) RA and OA iPSCs images after treatment of each TA concentration. (B)-(I) Real time PCR data expressed as mRNA levels of NMNAT3, PCNA, Ki67, p21, CDK4, CDK6, BAX and Bcl-2 after treatment with tannic acid (TA). (J)-(L) Annexin V assay data of RA and OA iPSCs after treatment of TA, each data show the population of early apoptotic, late apoptotic, viable cells. (N)-(Q) Flowcytometry analysis data of Annexin V assay. Real time PCR data and Annexin V data was presented as mean of OA iPSCs (n = 3) and RA iPSCs (n = 3). Data presented mean ± SEM. All data was analyzed by Student t-test. * means p < 0.05, ** means p < 0.01, *** means p < 0.001.

**Additional file 5: Table S1.** Sequences of primers used in RT-PCR. **Table S2.** Metabolites between OA FLS and RA FLS. **Table S3.** Metabolites between FLS and iPSC. **Table S4.** Metabolites between OA iPSC and RA iPSC. **Table S5.** Identified compounds using LC-Mass spectrometry.

## Data Availability

All datasets of this article are included within the article.

## Abbreviations

AMP: Adenosine monophosphate
FBS: Fetal bovine serum
FLS: Fibroblast-like synoviocyte
iPSC: Induced pluripotent stem cell
LysoPC: Lysophosphatidylcholine
NAM: Nicotinamide
OA: Osteoarthritis
RA: Rheumatoid arthritis
SN: Supernatant
