# Supplementary figures and images for: Metabolomic profiles of induced pluripotent stem cells derived from patients with rheumatoid arthritis and osteoarthritis
Source: Stem Cell Res Ther. 2019 Nov 15;10:319. doi: 10.1186/s13287-019-1408-5 (PMC6858676; doi:10.1186/s13287-019-1408-5)

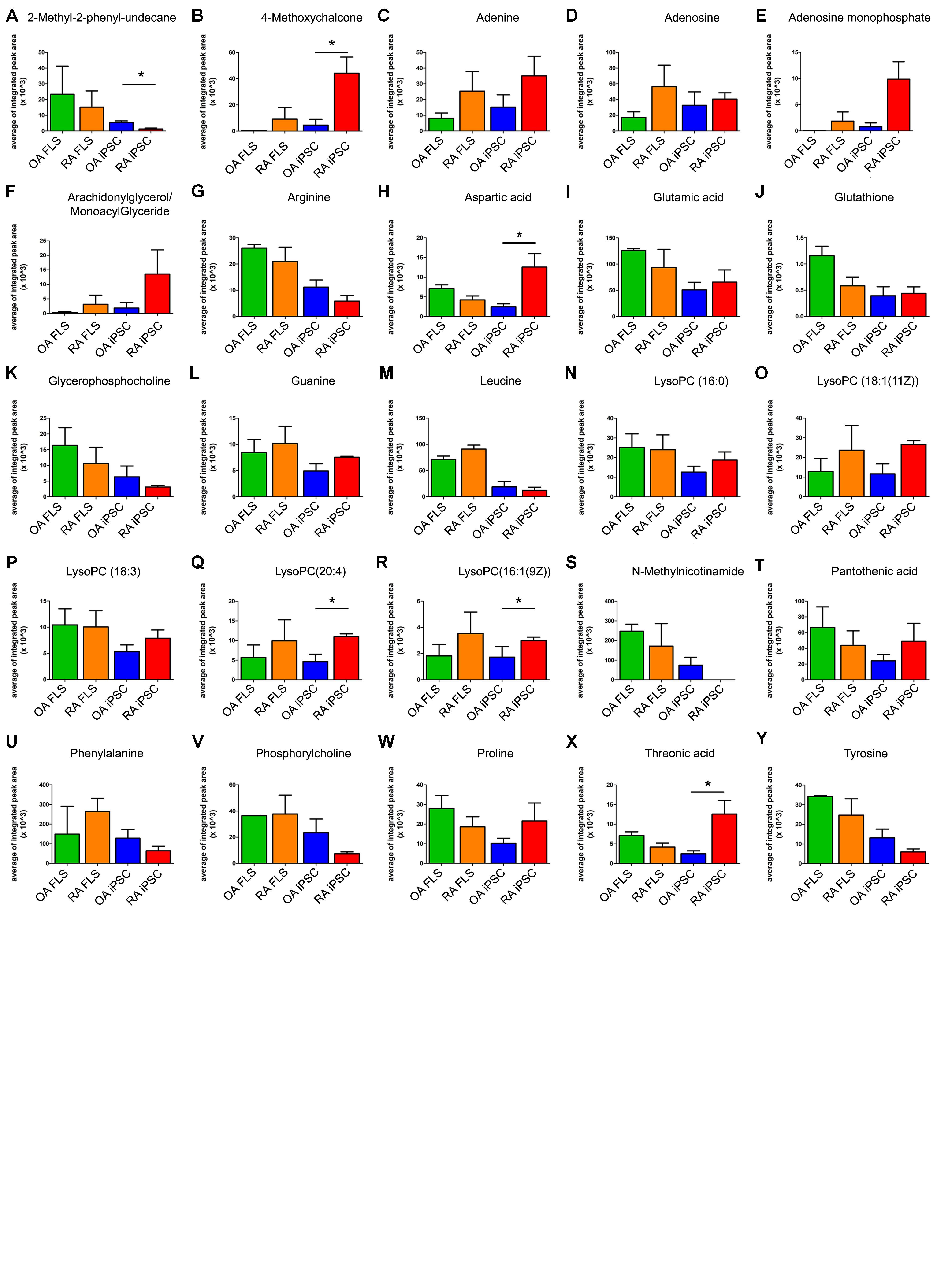

Supplement: Supplementary file 2 — Additional file 2. Fig S2. Statistical analysis of metabolites between OA, RA FLS cells and OA, RA iPSCs. Total of 26 metabolites including nicotinamide detected between OA, RA FLS cells and OA, RA iPSCs. Data presented mean ± SEM. All data analyzed by Student’s t-test. * means p < 0.05, ** means p < 0.01, *** means p < 0.001. [file 13287_2019_1408_MOESM2_ESM.jpg]

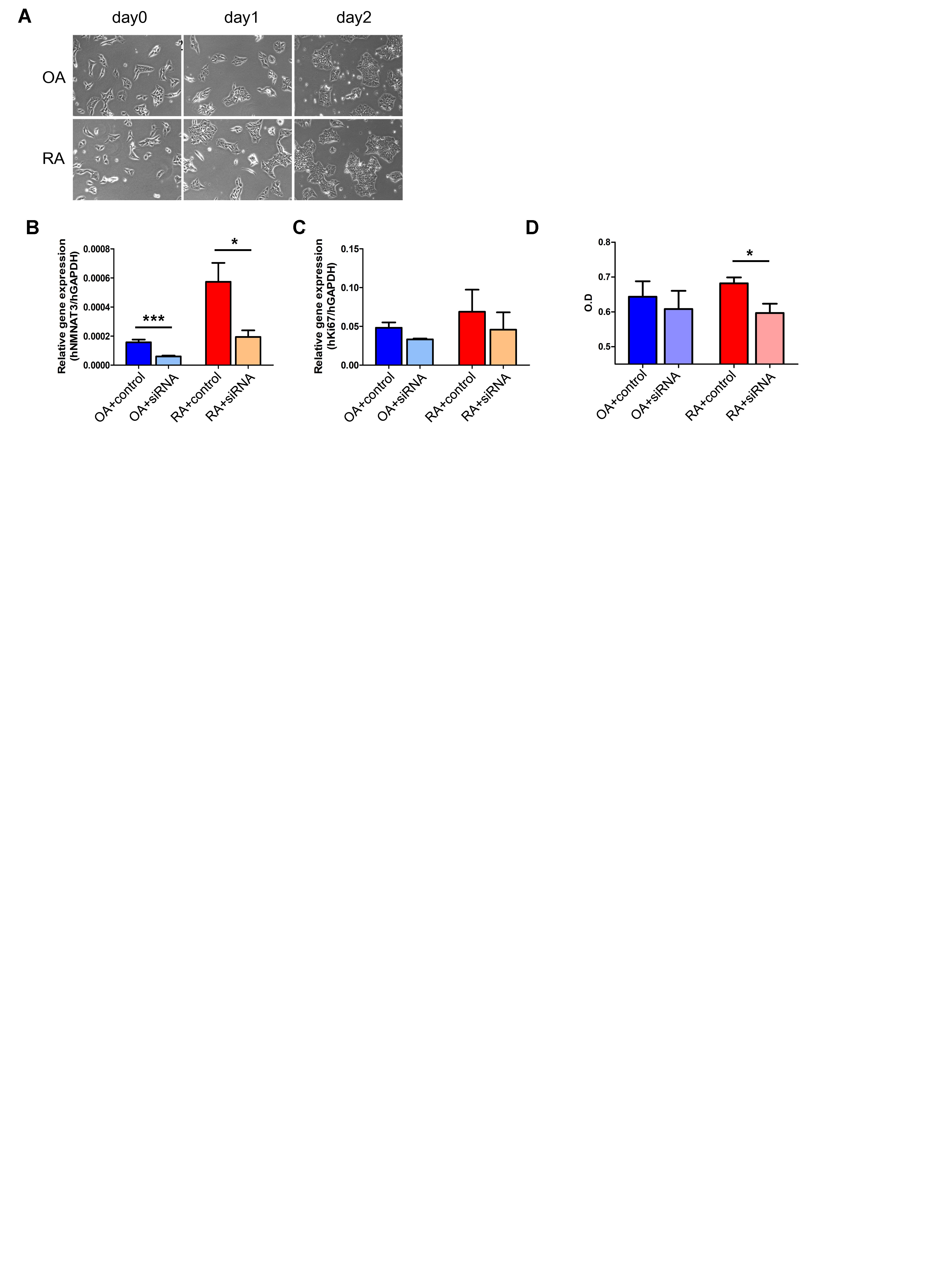

Supplement: Supplementary file 3 — Additional file 3. Fig S3. Inhibition of NMNAT3 shows that RA iPSC proliferation was reduced. (A) Cell proliferation images on a time dependent manner. (B)(C) Real time PCR data expressed as mRNA levels of NMNAT3, Ki67 after transfection of siRNA against NMNAT3. (D) CCK-8 assay of OA and RA iPSCs showing the difference of proliferation after transfection of siRNA against NMNAT3. Real time PCR and CCK8 assay data was presented as mean of OA iPSCs (n = 3) and RA iPSCs (n = 3). Data presented mean ± SEM. All data was analyzed by Student t-test. * means p < 0.05, ** means p < 0.01, *** means p < 0.001. [file 13287_2019_1408_MOESM3_ESM.jpg]

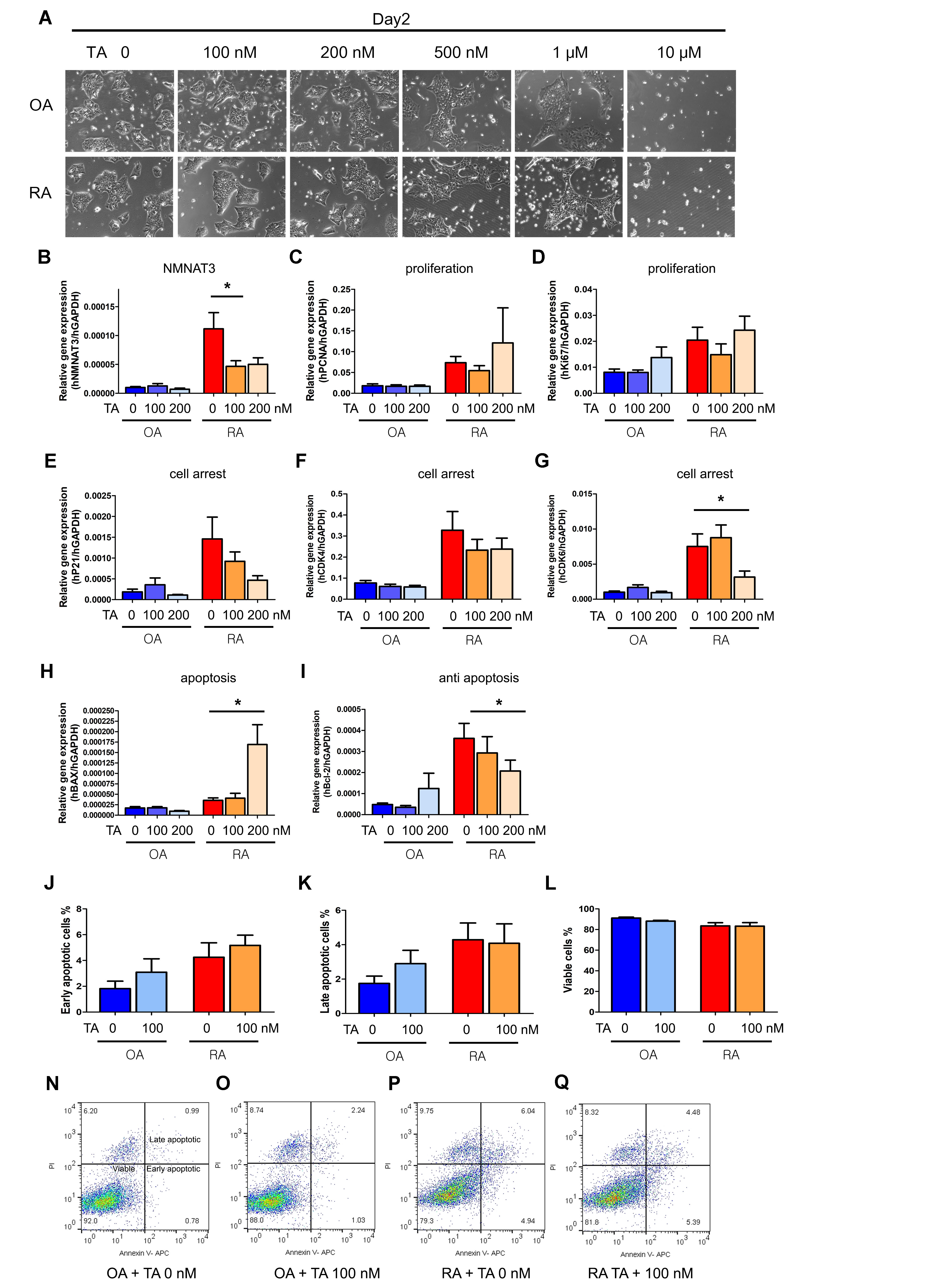

Supplement: Supplementary file 4 — Additional file 4. Fig S4. Inhibition of NMNAT3 after TA treatment. (A) RA and OA iPSCs images after treatment of each TA concentration. (B)-(I) Real time PCR data expressed as mRNA levels of NMNAT3, PCNA, Ki67, p21, CDK4, CDK6, BAX and Bcl-2 after treatment with tannic acid (TA). (J)-(L) Annexin V assay data of RA and OA iPSCs after treatment of TA, each data show the population of early apoptotic, late apoptotic, viable cells. (N)-(Q) Flowcytometry analysis data of Annexin V assay. Real time PCR data and Annexin V data was presented as mean of OA iPSCs (n = 3) and RA iPSCs (n = 3). Data presented mean ± SEM. All data was analyzed by Student t-test. * means p < 0.05, ** means p < 0.01, *** means p < 0.001. [file 13287_2019_1408_MOESM4_ESM.jpg]
