# Supplementary material for: Metabolomic profiles of induced pluripotent stem cells derived from patients with rheumatoid arthritis and osteoarthritis
Source: Stem Cell Res Ther. 2019 Nov 15;10:319. doi: 10.1186/s13287-019-1408-5 (PMC6858676; doi:10.1186/s13287-019-1408-5)
Supplement: Supplementary file 5 — Additional file 5: Table S1. Sequences of primers used in RT-PCR. Table S2. Metabolites between OA FLS and RA FLS. Table S3. Metabolites between FLS and iPSC. Table S4. Metabolites between OA iPSC and RA iPSC. Table S5. Identified compounds using LC-Mass spectrometry. [file 13287_2019_1408_MOESM5_ESM.docx]

Table S1. Sequences of primers used in RT-PCR

| **Target Name** | **Direction** | **Primer Sequence** | **Size** |
| --- | --- | --- | --- |
| NMNAT1 | Forward | TGGCACACAGCTTTTGTTTTG | 250 |
|  | Reverse | TCATCATGGCAGAACTTGCT |  |
| NMNAT2 | Forward | GGCACCGTCTCATCATGTGT | 271 |
|  | Reverse | CCCAAGATCTTGGCTGCAGT |  |
| NMNAT3 | Forward | CTACACCAAACAGCTGTGCC | 200 |
|  | Reverse | TTGTGCTGGTGCATCCGTAG |  |
| NAMPT | Forward | AGCTGTTCCTGAGGGCT | 284 |
|  | Reverse | AGTGAGCAGATGCTCCTATG |  |
| NNMT | Forward | ATTACAAGTTTGGTTCTAGG | 126 |
|  | Reverse | GGCCAGAGCCGATGTCAAT |  |
| OCT3/4 | Forward | ACCCCTGGTGCCGTGAA | 190 |
|  | Reverse | GGCTGAATACCTTCCCAAATA |  |
| SOX2 | Forward | CAGCGCATGGACAGTTAC | 321 |
|  | Reverse | GGAGTGGGAGGAAGAGGT |  |
| NANOG | Forward | AAAGGCAAACAACCCACT | 270 |
|  | Reverse | GCTATTCTTCGGCCAGTT |  |
| DPPA5 | Forward | CGGCTGCTGAAAGCCATTTT | 215 |
|  | Reverse | AGTTTGAGCATCCCTCGCTC |  |
| TDGF1 | Forward | TCCTTCTACGGACGGAACTG | 140 |
|  | Reverse | AGAAATGCCTGAGGAAAGCA |  |
| PCNA | Forward | CCTGCTGGGATATTAGCTCCA | 109 |
|  | Reverse | CAGCGGTAGGTGTCGAAGC |  |
| Ki67 | Forward | TGACCCTGATGAGAAAGCTCAA | 141 |
|  | Reverse | CCCTGAGCAACACTGTCTTTT |  |
| P21 | Forward | GTACCCTTGTGCCTCGCTCA | 119 |
|  | Reverse | CCGGCGTTTGGAGTGGTAGA |  |
| CDK4 | Forward | CTGACCGGGAGATCAAGGTA | 224 |
|  | Reverse | AGCCAGCTTGACTGTTCCAC |  |
| CDK6 | Forward | GCGCCTATGGGAAGGTGTTC | 160 |
|  | Reverse | TTGGGGTGCTCGAAGGTCT |  |
| BAX | Forward | TTCCGACGGCAACTTCAACT | 204 |
|  | Reverse | GGTGACCCAAAGTCGGAGAG |  |
| Bcl-2 | Forward | TCATGTGTGTGGAGAGCGTCAA | 179 |
|  | Reverse | CAGCCAGGAGAAATCAAACAGAGG |  |
| GAPDH | Forward | GAATGGGCAGCCGTTAGGAA | 414 |
|  | Reverse | GACTCCACGACGTACTCAGC |  |

Table S2. Metabolites between OA FLS and RA FLS

OA FLS > RA FLS

| ID | fold | p-value |  |
| --- | --- | --- | --- |
| Glutathione | 1.98 | 0.109 | ns |
| Aspartic acid | 1.68 | 0.151 | ns |
| Threonic acid | 1.68 | 0.151 | ns |
| Glycerophosphocholine | 1.55 | 0.517 | ns |
| 2-methyl-2-phenyl-undecane | 1.54 | 0.692 | ns |
| Pantothenic acid | 1.52 | 0.517 | ns |
| Proline | 1.50 | 0.339 | ns |
| N-methylnicotinamide | 1.44 | 0.648 | ns |
| Tyrosine | 1.39 | 0.440 | ns |
| Glutamic acid | 1.35 | 0.523 | ns |
| Arginine | 1.24 | 0.522 | ns |
| LysoPC (16:0) | 1.04 | 0.930 | ns |
| LysoPC (18:3) | 1.04 | 0.938 | ns |

OA FLS < RA FLS

| ID | fold | p-value |  |
| --- | --- | --- | --- |
| 4-methoxychalcone | 29.92 | 0.497 | ns |
| Adenosine monophosphate | 22.99 | 0.493 | ns |
| Arachidonylglycerol/monoacylglyceride | 10.62 | 0.530 | ns |
| Nicotinamide | 4.59 | 0.497 | ns |
| Adenosine | 3.31 | 0.350 | ns |
| Adenine | 3.15 | 0.362 | ns |
| LysoPC(16:1(9Z)) | 1.94 | 0.497 | ns |
| LysoPC (18:1(11Z)) | 1.84 | 0.571 | ns |
| Phenylalanine | 1.77 | 0.464 | ns |
| LysoPC (20:4) | 1.75 | 0.601 | ns |
| Leucine | 1.27 | 0.172 | ns |
| Guanine | 1.20 | 0.740 | ns |
| Phosphorylcholine | 1.04 | 0.949 | ns |

Table S3. Metabolites between FLS and iPSC

FLS > iPSC

| ID | fold | p-value |  |
| --- | --- | --- | --- |
| 1-methylnicotinamide | 5.40 | 0.034 | * |
| Leucine | 5.33 | < 0.0001 | *** |
| N-formylanthranilic acid | 3.78 | 0.005 | ** |
| Aminohydroxybutyric Acid  (4-Amino-3-hydroxybutyric acid) | 3.70 | 0.027 | * |
| L-Tryptophan | 3.39 | 0.019 | * |
| Pyrrolidonecarboxylic acid | 3.27 | 0.039 | * |
| Phenylpyruvic acid | 3.00 | 0.007 | ** |
| L-tyrosine | 2.99 | 0.007 | ** |
| Plasmenyl-PE 18:0 | 2.96 | 0.026 | * |
| Acetylcholine | 2.89 | 0.032 | * |
| L-threonine | 2.33 | 0.016 | * |
| cAMP | 2.22 | 0.042 | * |
| SM 34:1 | 2.20 | 0.011 | * |
| L-methionine | 2.10 | 0.004 | ** |
| Glutathione | 1.96 | 0.068 | ns |
| Guanine | 1.52 | 0.150 | ns |

FLS < iPSC

| ID | fold | p-value |  |
| --- | --- | --- | --- |
| Adenosine monophosphate | 7.92 | 0.035 | * |
| 4-methoxychalcone | 4.36 | 0.173 | ns |
| Arachidonylglycerol/MonoacylGlyceride | 3.85 | 0.315 | ns |
| Nicotinamide | 2.16 | 0.281 | ns |

Table S4. Metabolites between OA iPSC and RA iPSC

iPSC > RA iPSC

| ID | fold | p-value |  |
| --- | --- | --- | --- |
| N-methylnicotinamide | 399.20 | 0.139 | ns |
| 2-methyl-2-phenyl-undecane | 4.27 | 0.023 | * |
| Phosphorylcholine | 3.18 | 0.202 | ns |
| Tyrosine | 2.21 | 0.203 | ns |
| Glycerophosphocholine | 2.03 | 0.407 | ns |
| Phenylalanine | 2.00 | 0.270 | ns |
| arginine | 1.92 | 0.193 | ns |
| Leucine | 1.56 | 0.594 | ns |

OA iPSC < RA iPSC

| ID | fold | p-value |  |
| --- | --- | --- | --- |
| Adenosine monophosphate | 12.51 | 0.057 | ns |
| 4-methoxychalcone | 9.74 | 0.039 | * |
| Arachidonylglycerol/monoacylglyceride | 7.28 | 0.240 | ns |
| Niacotinamide | 5.27 | 0.013 | * |
| Aspartic acid | 5.07 | 0.045 | * |
| Threonic acid | 5.07 | 0.045 | * |
| LysoPC (20:4) | 2.37 | 0.032 | * |
| Adenine | 2.32 | 0.247 | ns |
| LysoPC (18:1(11Z)) | 2.28 | 0.052 | ns |
| Proline | 2.11 | 0.293 | ns |
| Pantothenic acid | 2.03 | 0.365 | ns |
| LysoPC(16:1(9Z)) | 1.73 | 0.217 | ns |
| Guanine | 1.54 | 0.136 | ns |
| LysoPC (16:0) | 1.49 | 0.298 | ns |
| LysoPC (18:3) | 1.48 | 0.277 | ns |
| Glutamic acid | 1.29 | 0.619 | ns |
| Adenosine | 1.24 | 0.694 | ns |
| Glutathione | 1.12 | 0.840 | ns |

Table S5. Identified compounds using LC-Mass spectrometry

| **Compound name** | **Exact mass** | **Adduct** | **Retention time** | **m/z** | **Related metabolism/pathway** | |
| --- | --- | --- | --- | --- | --- | --- |
| 2-methyl-2-phenyl-undecane | 190.171 | M+H | 9.37 | 191.179 | | NF-kB signaling |
| 4-methoxychalcone | 238.098 | M+H | 1.12 | 239.105 | | PI3K/Akt/mTOP pathway |
| Acetylcholine | 145.16 | M+H | 0.83 | 146.17 | | Acetylcholine pathway |
| Adenine | 135.054 | M+H | 1.15 | 136.061 | | Cellular respiration, protein synthesis |
| Adenosine | 267.096 | M+H | 1.17 | 268.104 | | Purine metabolism |
| Adenosine monophosphate | 347.062 | M+H | 1.17 | 348.07 | | Purine metabolism |
| cAMP | 329.06 | M+H | 1.14 | 330.07 | | Purine metabolism |
| Aminohydroxybutyric Acid | 119.12 | M+H | 1.08 | 120.064 | | GABA metabolism |
| Arachidonylglycerol/ | 378.274 | M+H | 13.2 | 379.281 | | Lipid metabolism, fatty acid metabolism |
| Monoacylglyceride |  |  |  |  |  |  |
| Arginine | 174.111 | M+H | 0.88 | 175.118 | | mTOR pathway |
| Aspartic acid | 133.037 | M+H | 1.1 | 134.044 | | Urea cycle and gluconeogenesis |
| Glutamic acid | 147.053 | M+H | 1.1 | 148.06 | | Citric acid cycle, Gluconeogenesis |
| Glutathione | 307.084 | M+H | 1.19 | 308.091 | | Glutathione metabolite pathway, |
|  |  |  |  |  |  | Glutamate metabolism |
| Glycerophosphocholine | 257.102 | M+H | 1.13 | 258.11 | | Glycerophospholipid metabolism, |
|  |  |  |  |  |  | Lipid metabolism pathway |
| Guanine | 151.049 | M+H | 1.18 | 152.056 | | De novo purine biosynthesis, |
|  |  |  |  |  |  | Purine salvage pathway |
| Formylanthranilic acid | 165.078 | M+H | 1.55 | 166.085 | | Tryptophan metabolism |
| Leucine | 131.095 | M+H | 1.16 | 132.102 | | Leucine metabolism-final end products include: acetyl-co A and acetoacetate |
| LysoPC (16:0) | 495.331 | M+H | 12.44 | 496.339 | | Phospholipid metabolism, |
|  |  |  |  |  |  | lipid metabolism, fatty acid metabolism |
| LysoPC (16:1(9Z)) | 493.314 | M+H | 11.7 | 494.321 | | Phospholipid metabolism, |
|  |  |  |  |  |  | lipid metabolism, fatty acid metabolism |
| LysoPC (18:1(11Z)) | 521.347 | M+H | 12.7 | 522.354 | | Phospholipid metabolism, |
|  |  |  |  |  |  | lipid metabolism, fatty acid metabolism |
| LysoPC (18:3) | 517.312 | M+H | 12.44 | 518.319 | | Phospholipid metabolism, |
|  |  |  |  |  |  | lipid metabolism, fatty acid metabolism |
| LysoPC (20:4) | 543.328 | M+H | 12.7 | 544.335 | | Phospholipid metabolism, |
|  |  |  |  |  |  | lipid metabolism, fatty acid metabolism |
| L-methionine | 149.051 | M+H | 1.17 | 150.057 | | Glycine and serine metabolism |
| L-threonine | 119.07 | M+H | 1.41 | 120.08 | | Glycine and serine metabolism |
| Nicotinamide | 122.048 | M+H | 1.13 | 123.055 | | De novo synthesis, |
| N-methylnicotinamide | 136.063 | M+H | 0.94 | 137.07 | | Metabolite of nicotinamide → glycolysis, Krebs cycle, gluconeogenesis |
| Pantothenic acid | 219.111 | M+H | 1.81 | 220.118 | | Beta-alanine metabolism, |
| plasmenyl-PE (phosphoethanolamine) 18:0 | 479.335 | M+H | 12.89 | 480.332 | | Phospholipid metabolism |
| Phenylalanine | 165.079 | M+H | 1.67 | 166.086 | | Amino acid synthesis |
| Phenylpyruvic acid | 164.047 | M+H | 1.19 | 165.054 | | Phenylalanine and tyrosine metabolism |
| Phosphorylcholine | 183.066 | M+H | 0.82 | 206.054 | | The glycine, serine and threonine metabolism pathways |
| Proline | 115.063 | M+H | 1.07 | 116.067 | | Methylation cycle |
| Pyrrolidonecarboxylic acid | 129.042 | M+H | 0.855 | 130.049 | | D-glutamine and D-glutamate metabolism |
| 34:1 SM (sphingomyelin) | 702.564 | M+H | 14.24 | 703.571 | | Lipid metabolism |
| Threonic acid | 136.038 | M+H | 1.1 | 137.046 | | Aldarate metabolism pathway |
| Tryptophan | 204.225 | M+H | 1.67 | 188.067 | | Tryptophan metabolism |
| Tyrosine | 181.073 | M+H | 1.2 | 182.081 | | Disulfiram action pathway, |
|  |  |  |  |  | | phenylalanine and tyrosine metabolism |
